# Benefits and Future of Clinical Autopsy: A Literature Review

**DOI:** 10.7759/cureus.93412

**Published:** 2025-09-28

**Authors:** Mohammad Abdurrahman Khan, Manisha Verma

**Affiliations:** 1 Department of Forensic Medicine and Toxicology, Hind Institute of Medical Sciences, Barabanki, Lucknow, IND; 2 Department of Dentistry, Hind Institute of Medical Sciences, Barabanki, Lucknow, IND

**Keywords:** autopsy, clinical autopsy, fetal autopsy, hospital autopsy, pathologist, pathology

## Abstract

Clinical autopsies are performed by pathologists in the hospital to diagnose the disease that has caused the death of a person when all antemortem attempts have become unsuccessful. Despite the cause of death being established antemortem, the clinical autopsy is carried out to study the process of disease in situ and to confirm the clinical diagnosis made by the physician. Thus, clinical autopsy enhances and improves the knowledge of medical science. Clinical autopsy is done on the request of family members of the deceased or by the physician; the consent of the deceased’s relatives is required. Before starting a clinical autopsy, all the documentary prerequisites should be fulfilled. Basic infrastructure required for clinical autopsy is a furnished room with adequate facilities for cold storage. Clinical autopsies are not restricted to being performed by a pathologist only. Any registered medical practitioner, especially a qualified pathologist, can carry out a clinical autopsy. After taking consent from the family members of the deceased, a needle, partial, or complete autopsy is performed. There are several benefits of clinical autopsies, such as providing benefits to the family members, improving the quality of diagnosis and treatment, training of pathologists and medical students, understanding the mechanism of sudden unexpected death, understanding the mechanism of fetal death, especially with genetic variation, etc. In the current scenario, there is a decline in the clinical autopsy rate despite several benefits of these autopsies. But clinical autopsy is still considered the gold standard as it helps in the measurement of treatment methods, clinical decision-making procedures, and diagnostic tools. For furnishing high-quality services, there should be sufficient pathologists having the required skill, well-trained and supporting staff, along with modern autopsy facilities. Besides this, there should be adequate support from the laboratory for providing a timely report of histopathology and other required tests.

## Introduction and background

The autopsy connects the cause of death to related pathologies and reveals the connection between the two. The term autopsy is a Greek word, “autopsia,” which means to see oneself. It is a combination of two words, one is autos, which means “self,” and the other is opsis, which means “eye” [1]. Autopsy refers to the examination of dead bodies for legal, medical, or systemic purposes [2]. The autopsy is mainly of two types: one is a forensic/medicolegal autopsy, and the other is a clinical/pathological/hospital autopsy. Medicolegal autopsies are performed in cases of violent, suspicious, or unexplained deaths to give answers to the various questions, such as the identity of the deceased, the circumstances of death, time of death, cause of death, etc., and thus benefit the law enforcement agencies to resolve the case. Medicolegal autopsy is performed by a forensic expert on the request of the inquest officer, and it does not require the consent of family members. Clinical autopsies are performed by pathologists in the hospital to diagnose the disease that has caused the death of the person when all the antemortem attempts become unsuccessful. Despite the cause of death having been established antemortem, the clinical autopsy is carried out to study the process of disease in situ and to confirm the clinical diagnosis made by the physician. Thus, clinical autopsy enhances and improves the knowledge of medical science. Clinical autopsy is done on the request of family members of the deceased or by the physician with the consent of a family member of the deceased [1,3]. Clinical autopsy not only determines the course of disease processes but also reveals the connection between symptoms and clinical diagnosis, and determines the efficacy of treatment, among other things [4]. Karl Rokitansky is considered the father of modern autopsy, as he conducted thorough examinations of each organ, regardless of pre-existing pathology. Rudolph Virchow applied the cell theory to develop the new area of cellular pathology. In the 20th century, the autopsy rate decreased remarkably, and the reason behind this seems to be recent advances in diagnostic technologies and the inadequacy of clinicians in making postmortem diagnosis [5]. There are many reasons for the decrease in autopsy rate, such as increased confidence in making antemortem diagnosis, lack of interest by clinicians, more complex laws related to the tissue procedure, and low priority given to autopsies by pathologists as they are already engaged in the increasing workload of cytology, biopsy, and surgical resections [6]. Other significant factors for declining autopsies are the risk of the autopsy procedure and the risk of exposure to diseases and infection for the pathologist. Moreover, the development of non-invasive, newer technologies, such as imaging and molecular technologies, tends to replace clinical autopsies [4].

The aim of this review article is to understand the clinical autopsy (to confirm the nature of the particular disease that has caused death, when all the ante-mortem efforts have failed, and to understand the pathology of disease, which has caused death, despite having established the diagnosis before death), the steps followed during the procedure of clinical autopsy, its significance, and the future of clinical autopsy.

## Review

Clinical autopsy

Documentary Prerequisites

Documentary prerequisites for a clinical autopsy include the present and past illnesses of the deceased, as mentioned on the case sheet. Requisition for clinical autopsy from the concerned physician with acknowledgment of specific doubt or dilemma, with the aim that better clinico-pathological correlation can be established. Consent from the family member of the deceased, mentioning all details such as the extent of autopsy and any required tissue or organ for investigation. Official letter from the person in charge of the hospital to the pathologist asking him to perform a clinical autopsy. The administration of the hospital must ensure that the death of the deceased was not a medicolegal case before the commencement of clinical autopsy [3].

Required Facilities

Basic infrastructure required for clinical autopsy is a furnished room with adequate facilities for cold storage. Other essential requirements for an autopsy room are adequate natural lighting, good ventilation, such as exhaust, a running water supply, and the autopsy area should be fly-proof, with good drainage of water, especially the autopsy table should have central drainage. Clinical autopsy should be done in natural light, as different hues of color are better in natural light [3].

Pathologist

Clinical autopsies are not restricted to being performed by a pathologist only. Any registered medical practitioner, especially a qualified pathologist, can carry out a clinical autopsy [3].

Procedure

All the provided clinical data of the deceased must be carefully studied. After taking consent from the family members of the deceased, a needle, partial, or complete autopsy is performed. External and internal examination is carried out thoroughly, noting all abnormalities as well as relevant negative findings. The clinical autopsy is preceded by making a Y-shaped incision starting from both shoulders, joining at the sternum, and descending to the pubic symphysis. The skin, along with the underlying tissue, is dissected to expose the chest and abdominal cavity. In clinical autopsy, internal examination is carried out more meticulously as it is more informative than the external examination. During the process of autopsy, the pathologist specifically looks for findings that match the clinical symptoms and signs described by the clinician so that a proper clinic-pathological correlation is achieved. One of the benefits of meticulous recording of all abnormalities during autopsy is to find out any other disease present in addition to the cause of death. During clinical autopsy, the relevant organ must be preserved as a routine for histopathological or microbiological examination. Finally, the cause of death is decided by gross examination of the organ and body tissue, and the result is intimated to the concerned clinician. Afterward, the death is confirmed by histopathological or microbiological findings of the preserved tissue [3].

Benefits of clinical autopsy

**Figure 1:**
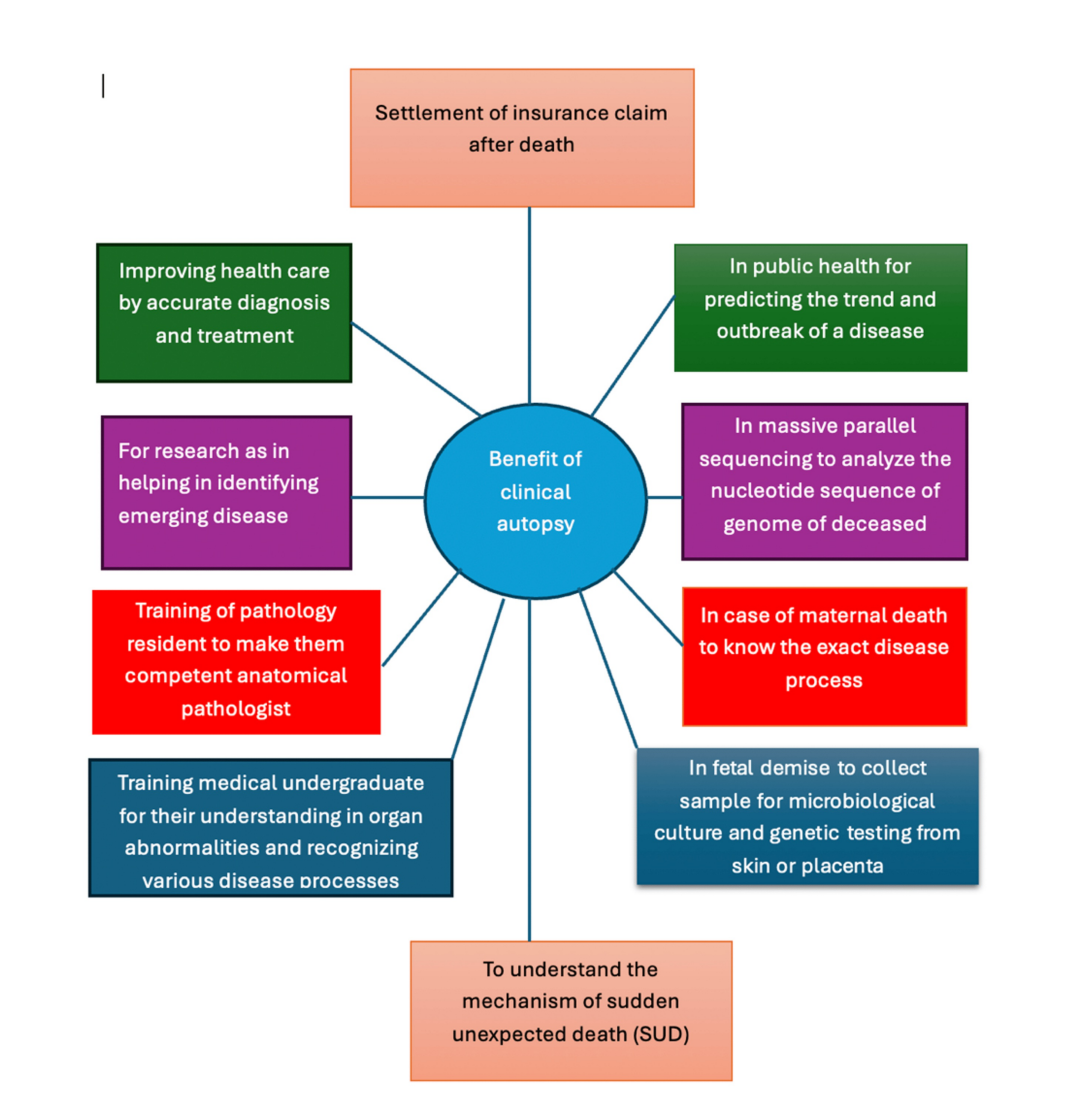
Benefits of clinical autopsy. The figure was created by Dr. Mohammad Abdurrahman Khan.

The benefits of clinical autopsies are discussed below (Figure 1).

Benefits to the Family Members, Including Compensation and Insurance Claims

Clinical autopsy can benefit the family members by disclosing heritable or genetic diseases and could alert other family members to early diagnosis and treatment. Clinical autopsy can also be helpful in detecting communicable infectious diseases, such as tuberculosis, which can affect other family members. By establishing the cause of death by clinical autopsy, it can reassure the family members that the care provided by them was adequate and did not contribute to the death of the deceased [7-9]. Clinical autopsy is also beneficial to uncover the death related to the occupational disease and hence helpful in providing compensation to family members. Clinical autopsy is also beneficial for settlement of insurance claims and hence provides death benefits for the family members [10].

Improving Quality in Diagnosis and Treatment

One of the important beneficial aspects of the autopsy is to improve the quality of healthcare by providing an accurate diagnosis and appropriate treatments. For improving the accuracy of the treatment, the role of clinical autopsy is revealed by the difference between the cause of death mentioned on the death certificate and the cause of death in the clinical autopsy. One study revealed 47% concordance between the autopsy finding and the death certificate [11].

Role of Clinical Autopsy in Research

One of the traditional and important roles of clinical autopsy is research. Human tissue research is of two types. The first type of human tissue research is not only to identify but also to fully characterize the various emerging diseases. Recent examples are Creutzfeldt-Jakob disease, description of amoebic encephalitis, and Reye’s syndrome [12]. Another example of a clinical autopsy-based study in Australia was the prevalence of alcohol-induced neurological disorders. There was the highest incidence of Wernicke-Korsakoff syndrome, which is due to severe deficiency of thiamine in chronic alcoholics. This study led to compulsory supplementation of thiamine in bread flour, and this action was validated by a follow-up clinical autopsy study, which revealed a fall in the prevalence of Wernicke-Korsakoff syndrome [12-14]. The second type of human tissue research is based on utilizing the emerging technologies along with standard methods for improving our knowledge in recognizing the disease. An example of this type of human research is the application of recombinant DNA technologies for improving our knowledge and understanding of various types of bowel cancer. Though a major part of this work could be achievable with sectioning of tissue during life, the clinical autopsy is the key source of brain tissue for molecular research into neurodegenerative disorders, such as amyotrophic lateral sclerosis and Parkinsonism, and the source of brain tissue is the autopsy [15,16].

Role of Clinical Autopsy in the Training of Pathologists

The Association of Pathology Chairs (APC) had organized a meeting in 2014, where some pathologists were in favor of discontinuing autopsy training from the training curriculum of pathology residents to give extra months of training in recent disciplines, such as molecular genetics and informatics. Concurrently, the American Board of Pathology (ABP) received complaints that newly joined pathologists with a certificate of pathological anatomy were not competent in carrying out an autopsy when requested. In response to this, the APC formed the Autopsy Working Group to determine the role of autopsy training in the pathology resident training curriculum. After thorough examination, the Autopsy Working Group submitted its final report to the APC. The report stated that autopsy training is an essential part of the training curriculum of pathology residents, and this training can transform a newly graduated medical resident into a competent anatomical pathologist who can perform clinical autopsies. The standardized and regular training of the beginner pathologist could improve the technique of postmortem examination. Cases available for clinical autopsies give great opportunities to the beginner pathologist to correlate the findings of the clinical autopsy with information provided from physical examination, laboratory investigation, and radiological examination. The more time spent by the novice pathologist inside the autopsy room, the more the development of a comprehensive anatomic-clinical view. It will be possible for a novice pathologist to enhance knowledge through each clinical autopsy case, including macroscopic and microscopic findings of various diseases. Hence, clinical autopsy brings the elementary data for ameliorating the quality of medical care and training [17].

Role of Clinical Autopsy in Medical Training

Detailed and extensive knowledge of the human body is an essential part of all phases of the medical curriculum. Autopsies can provide an opportunity for medical students, where they can acquire knowledge about the abnormalities in organs, enhance their understanding of asymptomatic or commencing lesions, understand the aging of human bodies, recognize the various diseases through the response of the body, and establish the actual mechanism for the cause of death. Visiting the autopsy room should be a curricular activity during medical education training. Enhancing knowledge through learning various topics, such as physiology, anatomy, embryology, genetics, histology, and immunology, could be achieved by visiting and participating in autopsies. It would be better for medical students of each year to attend the autopsy room in groups, where various responsibilities and activities may be different but integrated. In such circumstances, the pathologist may act as a physician with the role of performing intersection and integration among medical students and the medical team involved in laboratory and imaging investigation. To gain medical knowledge and skill, the most appropriate triad is patient care, diagnostic tools, and the clinical autopsy. This will provide a learning platform and exceptional knowledge to the young medical graduate, thus encouraging the idea of a holistic view of the patient and integrating the acquired medical knowledge from an early age [18].

Role of Clinical Autopsy in Sudden Unexpected Death

Sudden unexpected death (SUD) is death occurring in a healthy individual from a natural cause within an hour of the onset of symptoms. Thus, it is an unexpected fatal event that could not be predicted and often goes unwitnessed. SUD is sudden, which occurs within a few hours of the first manifestation of a disease, which is unexpected from medical history [18,19]. SUD includes sudden intrauterine death (SIUD), sudden infant death (SIDS), and sudden unexpected death of the young (SUDY). SUD can take place at any time without any warning and is one of the serious problems of public health, having an immense emotional and economic impact on family members of the deceased [20]. In SIDS, meticulous examination of anatomical structure, especially the cardiac autonomic conduction system, remains neglected. Studies by Ottaviani et al. and Ottaviani & Buja revealed abnormalities in the cardiac autonomic conduction system and brainstem regulatory nuclei in SIUD and SIDS, which gives strong evidence to support meticulous clinical autopsies in each case of SUD [21,22]. Such meticulous autopsies include extensive examination of the cardiac autonomic conduction system, where the cardiac electrical impulse starts, and the brainstem regulatory nuclei that regulate respiratory, circulatory, and arousal activities of an individual [23]. The critical finding revealed during clinical autopsy of SUD gives facts to the surviving family member of the deceased, particularly about genetic defect or abnormalities, and also enables making correlation with various risk factors, and also to find out the possible trigger that may interact with inherited genetic variation [18].

Clinical Autopsy in Fetal Death, Especially With Genetic Variation

Fetal demise during the first trimester or in the early 2nd trimester of the pregnancy brings an opportunity to the pathologist for fetal autopsy. Clinical autopsy of a fetus is especially useful in the gestational period of up to 15 weeks [24]. It is recommended to carry out meticulous examination of the fetus and placenta in a fresh condition before fixation. During autopsy, besides dissection of the fetus, a sample should be collected not only for microbiological culture but also for genetic testing via skin or placenta. The reason for fetal loss occurring during the first trimester or the early 2nd trimester is chromosomal abnormalities. It is also necessary to collect specimens for histopathological examination of the vital organs of the body. Before starting the clinical autopsy of the fetus, it is advisable to take a photograph and a radiograph of the fetus. Since genetic diseases are congenital, they may be expressed much after birth, even sometimes in the adult stage. However, the genetic disorder that is expressed early, especially during the period of embryogenesis, tends to be detected more during clinical autopsy. The accurate identification of the congenital anomalies and their genetic examination are one of the important steps of reproductive counseling and thus reassure the family members of the deceased who otherwise experience helplessness [18].

Clinical Autopsy on Maternal Mortality

To reduce maternal mortalities, it is essential to understand the etiologies of maternal death. The main causes of maternal mortalities in India are hemorrhage (47.2%), pregnancy-related infection (12%), hypertensive disorder (6.7%), abortion (4.9%), and some other direct and indirect causes [25]. Many such maternal mortalities can be avoided if diagnosed and managed early [26]. There are certain conditions where maternal mortality occurs at home or the patient dies just reaching the emergency room in a very serious condition; in such situations, a meticulous clinical autopsy should be carried out to find out the cause of maternal death; accordingly, an action plan should be taken [18,27]. Many clinical conditions of pregnancy could not be suspected during maternal evaluation, even in a good hospital with good prenatal care. Such clinical conditions are amniotic fluid embolism, myocarditis, cardiomyopathies, congenital heart disease, and primary pulmonary disease. In such a scenario, it is important to perform a systemic clinical autopsy to identify such direct or indirect causes of maternal mortality so that it could be beneficial to assess the possibility that maternal death was preventable and hence it may play a role in the prevention plan of maternal mortality in public health strategies [18].

Sequence Analysis of the Deceased’s Genomic as a Routine Part of Clinical Autopsy

Massively parallel sequencing is a technology by which the nucleotide sequence of the various regions of DNA can be analyzed. Eventual bioinformatic and methodological improvement enables sequencing of a larger region of genomic DNA with comparatively easy and quick speed. One of the important characteristics of genomic DNA is that it is inherited via the germline and remains intact within dead cells over a period of time, and can be easily extracted from any of the cells. It can also be extracted if it is already fixed in formalin or even embedded in paraffin. The main goal of DNA sequencing from the patient is to diagnose the disease and recognize the target region for the drugs that may neutralize or counteract the effects produced by inherited mutations in specific genes, such as cancer. This prototype is known as precision medicine or personalized medicine, so it is an emerging approach for treatment and prevention that is based on individual variability in genes, lifestyle, and environment. Genomic DNA screening as a routine part of clinical autopsy would be based on a practiced policy that clinical autopsies are not limited to a particular organ. All organs of the deceased should be examined extensively, and specimens are collected randomly from the tissue of each organ for microscopy, in addition to any macroscopic lesion that is observed during clinical autopsy. Besides gross and microscopic examination of the various organs during clinical autopsy, blood and tissue culture are analyzed for microorganisms, body fluid for biochemical abnormalities, and X-ray for body parts. Among these procedures, some may be restricted to cases having gross lesions or a certain history, but for others, it is a routine part of all clinical autopsies [28,29].

Role of Clinical Autopsy in Public Health

Clinical autopsy can help in predicting the trend of disease and its outbreak. The officials of public health can use the findings of the autopsy to develop policies that could not only reduce the transmission of disease but also decrease the mortality rates [30].

Future aspects of clinical autopsy

Despite a decrease in autopsy rate, postmortem examination remains the gold standard for determining the cause of death of a person. As revealed by several studies, in 30% of cases, death was not correctly identified, despite intensive patient care and precise diagnostic techniques [31,32]. In today’s scenario, there is a need to develop newer technology that should act as an alternative to clinical autopsies.

Virtopsy

One such hopeful alternative is a CT image, which acts as an adjuvant for determining the cause of death during clinical autopsy, a practice well known as virtopsy [33]. CT image and autopsy are complementary procedures [34]. CT image of hypertensive pneumothorax, and even in certain conditions such as aortic dissection succeeded by cardiac tamponade, can be identified by a CT image during clinical autopsy. Use of virtopsy for identifying fractures in deceased with polytrauma or identifying the firearm bullet and its location is one of the valuable benefits during autopsy. This prototype of postmortem examination, i.e., use of CT image during clinical autopsy, is an encouraging future in which both radiologist and pathologist can work jointly as specialists to determine the cause of death and thus evade the entire procedure of autopsy [18,35].

Minimally Invasive Autopsy

With the embodiment of precision medicine, particularly in the treatment of patients having carcinoma, pathology has a pivotal role in regulating the therapies for cancer with molecular assistance for genes that are mutated. The minimally invasive autopsy (MIA) is an outstanding option that is guided by imaging examinations [18]. MIA would play a pivotal role in the field of clinical research for the retrieval of the targeted fragments of the tumor from metastasis. MIA has several benefits, such as being more economical than the conventional autopsy. MIA can be carried out in a shorter duration after demise and can be performed at the bedside just after the death of the patient. In addition, MIA is comparatively less invasive than the routine autopsy procedure, and hence easier to obtain consent from family members. MIA is conclusive in certain cases, such as direct study of diseased organ, confirmation of disseminated malignant neoplasia, or when there is a high risk for acquiring infection in the whole team, as in COVID-19 death [36]. MIA can be used for taking specimens, which can be frozen for refined methodologies and techniques used to investigate genetic and molecular pathology [18,37]. In the present scenario, with the growth in advancement of medical sciences and imaging, it seems to decrease the need for autopsy. But the current worldwide pandemic exposed just the opposite. During the period of the COVID-19 pandemic, either no autopsy or only a few autopsies were carried out, which resulted in the world's struggle in treating COVID-19 patients because the pathological basis of the disease was not known. During the COVID-19 pandemic, a large number of patients acquired the COVID-19 disease simultaneously with a wide spectrum of several aspects of the disease, which makes it difficult not only to understand but also to predict the course of COVID-19. From the beginning of the COVID-19 pandemic until now, several research papers have been published, but still, the actual disease process is not well known. Clinical autopsy can play a pivotal role in addressing such complex disease concerns in an integrated approach [38].

Reasons for declining autopsy rate

As revealed in many studies, the decline in clinical autopsy is due to an adverse public perception of clinical autopsy [12]. One study in the United Kingdom revealed that after the “organ retention scandal,” when asked for consent for clinical autopsy, only 43.6% of family members of the deceased granted the permission [39]. If the family members of the deceased are properly informed and talk in a contemplative manner, there will be a substantial degree of selfless support from the community for research, based on clinical autopsy [40]. One more important aspect of decreasing autopsy is the reduced rate of requests by clinicians for clinical autopsy. One study revealed that clinicians requested only in 6.2% of cases for clinical autopsy, even though the percentage of consent is 43.4% [39]. More studies recommend that the combined effort of the concerned hospital, clinician, and pathologist may increase the rate of clinical autopsy by approximately 50% [12]. The role of a pathologist in seeking consent for clinical autopsy cannot be excluded. One study revealed that less than one-third of pathologists are involved in seeking consent from family members of the deceased [12].

Out of several reasons, one suggested for why hospitals and clinicians do not encourage clinical autopsies is the fear that if any clinically significant misdiagnosis is ruled out [41]. Costache et al. found a total correspondence rate (agreements between antemortem diagnosis and diagnosis after clinical autopsies) of 55.36%, partial correspondence rate (agreement of one or two diagnoses) of 26.79%, and total disagreement rate of 17.86% [5]. One study in Australia reported that there is a lack of autopsy facilities and that funding is not provided to the staff involved in autopsy services. There was no significant view regarding the funding of autopsies [42]. In the private sector, clinical autopsies are still not funded [12]. The Workforce Shortages Survey of the Royal College of Pathologists of Australasia found that the contribution of pathologists should also be considered in the decline in the rate of clinical autopsy. High workload and workforce shortage for anatomical pathologists make their limited availability for performing clinical autopsy [43]. One study in their survey revealed a continuous increase in the rate of perinatal autopsies. A factor behind this may be that perinatal and pediatric pathologists are more likely to practice in the tertiary care hospital and hence carry out such difficult perinatal autopsies as they have the training, special skill, and interest (Table 1) [44].

**Table 1:** Reasons for the decline in autopsy rates as described in various studies.

S. No.	Author	Study name	Reasons
1.	Royal College of Pathologists of Australasia Autopsy Working Party [12]	The decline of the hospital autopsy: a safety and quality issue for healthcare in Australia	Adverse public perception of clinical autopsy.
2.	Burton et al. [39]	Necropsy practice after the "organ retention scandal": requests, performance, and tissue retention	Reduced rates of requests by clinicians for clinical autopsy after the organ retention scandal.
3.	Shojania et al. [41]	Changes in rates of autopsy-detected diagnostic errors over time: a systematic review	Hospitals and clinicians do not encourage clinical autopsies due to the fear that any clinically significant misdiagnosis might be ruled out.
4.	Costache et al. [5]	Clinical or postmortem? The importance of the autopsy; a retrospective study	Total correspondence rate (agreements between antemortem diagnosis and diagnosis after clinical autopsies) of 55.36%, the partial correspondence rate (agreement of one or two diagnoses) was 26.79% and the total disagreement rate of 17.86%.
5.	Start et al. [42]	Funding the clinical autopsy	Lack of autopsy facilities and funding was not provided to the staff involved in autopsy services. There was no significant view regarding the funding of autopsies.
6.	Royal College of Pathologists of Australasia Autopsy Working Party [12]	The decline of the hospital autopsy: a safety and quality issue for healthcare in Australia	In the private sector, clinical autopsies are still not funded.
7.	Royal College of Pathologists of Australasia [43]	Workforce Shortages Survey 2003	High workload and workforce shortage for anatomical pathologist make their limited availability for performing clinical autopsy.
8.	Gordijn et al. [44]	Value of the perinatal autopsy: critique	Continuous increase in the rate of perinatal autopsies.

Steps to stop the decline and increase the rate of autopsy

There should be sufficient pathologists having the required skill, along with an interest in clinical autopsy, to furnish high-quality service. There should be well-trained and supporting staff along with modern autopsy facilities. Besides this, there should be adequate support from the laboratory for providing a timely report of histopathology and other required tests. After completion of the clinical autopsy, the final written autopsy report should be timely, accurate, sufficient, and easily understandable. Clinicopathological correlation must be established after completion of clinical autopsy.

There should be a provision for providing information to the family members and community regarding the autopsy, so that they can understand the illness of their deceased. This will surely build confidence and trust in the health sector; this will change the traditional mindset of autopsy being a violation of an individual’s integrity [12]. Although it is difficult, it is not impossible to implement the above-mentioned measures, as it requires multi-step cooperation in health sectors, such as providing quality service by a pathologist, a healthy relationship between the pathologist and healthcare worker providing care to the concerned patient, and support from the hospital administration. Community involvement will play a significant role in the success of increasing clinical autopsies. To enhance the value and role of clinical autopsy in the community, it is recommended to provide high-quality media-based information to the community [45].

## Conclusions

Clinical autopsy can benefit the family members by disclosing heritable or genetic diseases and could alert other family members to early diagnosis and treatment. Clinical autopsy is also beneficial to uncover the death related to the occupational disease and hence helpful in providing compensation to family members. It improves the quality of healthcare by providing accurate diagnoses and appropriate treatments. It will be possible for a novice pathologist to enhance knowledge through each clinical autopsy case, including macroscopic and microscopic findings of various diseases. Hence, clinical autopsy brings the elementary data for ameliorating the quality of medical care and training. Autopsies can provide an opportunity for medical students where they can acquire knowledge about the abnormalities in organ, enhance understanding about asymptomatic or commencing lesions, can understand the aging of human bodies, recognize the various diseases through response of the body, and could be able to establish the actual mechanism for the cause of death. Clinical autopsy may be beneficial in sudden, unexpected death to understand the mechanism involved in such death. Clinical autopsy is also beneficial in fetal death, if death occurs in the first trimester, and is associated with any genetic variation. In the current scenario, there is a decline in the clinical autopsy rate despite several benefits of these autopsies. But clinical autopsy is still considered the gold standard as it helps in the measurement of treatment methods, clinical decision-making procedures, and diagnostic tools.
